# Phenolic Constitution, Phytochemical and Macronutrient Content in Three Species of Microgreens as Modulated by Natural Fiber and Synthetic Substrates

**DOI:** 10.3390/antiox9030252

**Published:** 2020-03-20

**Authors:** Marios C. Kyriacou, Christophe El-Nakhel, Antonio Pannico, Giulia Graziani, Georgios A. Soteriou, Maria Giordano, Mario Palladino, Alberto Ritieni, Stefania De Pascale, Youssef Rouphael

**Affiliations:** 1 Department of Vegetable Crops, Agricultural Research Institute, 1516 Nicosia, Cyprus; m.kyriacou@ari.gov.cy (M.C.K.); soteriou@ari.gov.cy (G.A.S.); 2 Department of Agricultural Sciences, University of Naples Federico II, 80055 Portici, Italy; Nakhel_Christophe@hotmail.com (C.E.-N.); antonio.pannico@unina.it (A.P.); maria.giordano@unina.it (M.G.); mario.palladino@unina.it (M.P.); depascal@unina.it (S.D.P.); 3 Department of Pharmacy, University of Naples Federico II, 80131 Naples, Italy; giulia.graziani@unina.it (G.G.); alberto.ritieni@unina.it (A.R.)

**Keywords:** agave fiber, capillary mat, cellulose sponge, coriander, carotenoids, flavonoids, kohlrabi, nitrate, Orbitrap LC-MS/MS, pak choi, phenolic compounds

## Abstract

The present study examined the modulatory effects of natural fiber substrates (agave fiber, coconut fiber and peat moss) and synthetic alternatives (capillary mat and cellulose sponge) on the nutritive and phytochemical composition of select microgreens species (coriander, kohlrabi and pak choi) grown in a controlled environment. Polyphenols were analyzed by UHPLC-Q-Orbitrap-HRMS, major carotenoids by HPLC-DAD, and macro-minerals by ion chromatography. Microgreens grown on peat moss had outstanding fresh and dry yield but low dry matter content. Natural fiber substrates increased nitrate and overall macro-mineral concentrations in microgreens compared to synthetic substrates. The concentrations of chlorophylls, carotenoids and ascorbate were influenced primarily by species. On the contrary, variability in polyphenols content was wider between substrates than species. Out of twenty phenolic compounds identified, chlorogenic acid and quercetin-3-*O*-rutinoside were most abundant. Hydroxycinnamic acids and their derivatives accounted for 49.8% of mean phenolic content across species, flavonol glycosides for 48.4% and flavone glycosides for 1.8%. Peat moss provided optimal physicochemical conditions that enhanced microgreens growth rate and biomass production at the expense of phenolic content. In this respect, the application of controlled stress (eustress) on microgreens growing on peat moss warrants investigation as a means of enhancing phytochemical composition without substantial compromise in crop performance and production turnover. Finally, nitrate deprivation practices should be considered for microgreens grown on natural fiber substrates in order to minimize consumer exposure to nitrate.

## 1. Introduction

Over the past three decades, several researchers have tried to boost the content of vegetables in phytochemicals (e.g., ascorbate, carotenoids, glucosinolates, polyamines, polyphenols) through plant breeding and biotechnology, but so far, they met limited commercial success due to safety issues [1]. Therefore, searching for nutrient-dense vegetables through the manipulation of phytochemicals by environmental (air temperature, light quality, intensity and photoperiod) and innovative crop management practices (growing media, nutrition and biofortification) represents a promising and balanced approach between safety, cost and effectiveness [2,3]. Microgreens (i.e., edible seedlings of herbs, grains and vegetables), also known as vegetable confetti, are an emerging class of specialty crop that have gained increasing popularity among consumers, urban farmers, food technologists and nutritionists due to their fortified phytochemical composition, accumulated in the two fully developed cotyledons and the first true leaves, compared to their mature counterparts [4,5,6,7,8,9]. In addition to their potential nutritional and functional benefits, microgreens production presents the following advantages: (i) short cultivation cycle, (ii) all-year round production, (iii), ease of cultivation, (iv) suitability for indoor farming technology, (v) high potential returns/profitability for producers with an estimated value of 30–50 $ per pound and (vi) higher sustainability compared to growing mature herbs and vegetables, offering a small footprint in terms of space, water and fertilizers [8,10,11]. Moreover, the brief production cycle combined with rigorous potential returns for farmers makes microgreens a prominent controlled environment candidate crop [8].

Although several studies revealed that variation in microgreens’ content of bioactive compounds is based on several pre-harvest factors such as genetic material (i.e., species), conditions of cultivation and light parameters (i.e., spectral quality and intensity), additional variables have also been implicated in shaping microgreens’ nutritive and phytochemical composition, including nutrition/biofortification and choice of growth medium [4,10,12,13,14,15,16,17,18,19,20,21]. Notwithstanding the short crop cycle, special attention must be placed on the selection of growth media for microgreens, which represents one of the most important factors in the production process influencing microgreens quality [22]. Among common growing media used to produce microgreens, peat-based media come first, followed by coconut coir and synthetic fibrous media [10]. Recently, natural fiber-based media such as burlap, jute, cotton, cellulose pulp, kenaf and hemp fibers have gained increasing popularity in the microgreens industry, since they represent natural, sustainable and cheap alternatives for microgreens production [10,22]. However, few studies have examined the effects different types or combinations of media have on the yield of variable microgreens genotypes [22,23]. Muchjajib et al. [23] demonstrated that the 1:1 mixture of coconut coir dust and peat provided the highest yield for spinach microgreens, while the 1:1 mix of coconut coir dust with sugarcane filter cake exhibited the maximum yield for radish, mustard, krathin and kangkong microgreens. Additionally, Di Gioia and co-workers [22] demonstrated that organic (peat) and recycled fibrous materials (textile and jute-kenaf fibers) supported a fresh yield of rapini (*Brassica rapa* L.) higher by 15% to that on the synthetic fibrous material Sure-to-Grow^®^, which is marketed as a substrate consisting of plastic fibers with high water retention capacity. The same authors reported that peat-grown rapini microgreens had the highest population of *Escherichia coli* and *Enterobacteriaceae*, which was not detected on *B. rapa* microgreens grown on other substrates. However, scientific information on phytochemical profiles, and how these bioactive secondary metabolites respond to organic, synthetic and novel by-product substrates in emerging microgreens like coriander, kohlrabi and pak choi is completely missing.

Microgreens are currently considered among the five most profitable crops globally, along with mushrooms, ginseng, saffron and goji berries. Therefore, developing species-specific growth media to support year-round production and to enhance valuable antioxidant components is affordable and of utmost importance for the microgreens industry; particularly as the latter is characterized by high investment in technology (e.g., lighting and growth substrates) driven by the necessity to cultivate novel, highly fortified microgreen species. Considering the above considerations, the present study aimed (i) to characterize and elucidate the modulatory effects of natural fiber substrates (agave fiber, coconut fiber and peat moss) and synthetic substrates (capillary mat and cellulose sponge) on the nutritional and phytochemical composition (minerals, nitrate, chlorophylls, target carotenoids, ascorbate and polyphenols) of select microgreens (coriander, kohlrabi and pak choi), and (ii) to appraise possible clustering patterns underscoring microgreens composition and substrates physico-chemical characteristics.

## 2. Materials and Methods 

### 2.1. Reagents and Standards Preparation

The reagents methanol and formic acid (LC-MS grade) were purchased from Merck KGaA (Darmstadt, Germany); β-carotene, chicoric acid, chlorogenic acid, caffeic acid, catechin, epicatechin, ferulic acid, lutein, quercetin-3-*O*-glucoside, rosmarinic acid, rutin and vitexin standards were purchased from Sigma (St. Louis, MO, USA) and 3,5-di-*O*-caffeoyl quinic acid, kaempferol-7-*O*-glucoside, kaempferol-3-*O*-rutinoside and quercetin-3-*O*-galactoside from Extrasynthese (Genay, France). A Milli-Q Gradient A10 water purification system (Merck Millipore, Darmstadt, Germany) was used to produce ultrapure water. The standards were 98% pure, prepared with methanol to form 1 mg mL^−1^ initial stock solutions, while for lutein and β-carotene, chloroform was used to prepare the stocks of 1 mg mL^−1^ as well. Then, in order to acquire standard calibration curves of 0.01–5.0 mg L^−1^ span, individual standard stock solutions were combined to prepare multiple standards stock solutions by applying further dilutions made with methanol.

### 2.2. Plant Material and Climate Chamber Conditions

Three microgreen species, kohlrabi (*Brassica oleracea* L. var. *gongylodes*; Purple Vienna, Condor Seed Production, Yuma, AZ, USA), pack choi (*Brassica rapa* L. subsp. *chinensis*; Red Wizard F1, CN Seeds Ltd., Pymoor, Ely, Cambrigeshire, UK) and coriander (*Coriandrum sativum* L.; Micro Splits, CN Seeds Ltd., Pymoor, Ely, Cambrigeshire, UK), were sown in 5 different substrates: agave fiber (Sisal Fibre, Imola, Italy), capillary mat (Aquamat capillary matting, Premier Netting, Norfolk, UK), cellulose sponge (Spontex SAS, Colombes, France), coconut fiber (Sisal Fibre, Imola, Italy) and peat moss (Floragard, Oldenburg, Deutschland). Sowing density was 60,000, 63,000 and 46,000 seeds m^−2^ for kohlrabi, pack choi and coriander, respectively. Hundred-seed weight was 0.320, 0.240 and 0.684 g for kohlrabi, pack choi and coriander, respectively.

Experiments were carried out in a climate chamber (Panasonic MIR-554, Gunma, Japan) at the Agricultural Research Institute (ARI), Nicosia, Cyprus, using Light Emitting Diode (LED) panel units (K5 Series XL750, Kind LED, Santa Rosa, CA, USA) procuring a photosynthetic photon flux density (PPFD) of 300 ±10 µmol m^−2^ s^−1^ and a spectral composition matching the optimal absorption spectrum of photosynthesis. Seed germination occurred in darkness at 24 °C and 100% relative humidity. Day/night temperatures during the growth cycle were set at 22/18 ± 1 °C with a 12 h photoperiod and a relative air humidity of 65%/75% ± 5%. These levels of relative humidity ensured that the development of potentially harmful mycotoxins-producing molds was not observed in our experiments. The substrates were placed in plastic trays (14 × 19 × 6 cm: W × L × D). Fertigation was applied everyday manually by means of a laboratory beaker. The nutrient solution corresponded to a quarter-strength modified Hoagland formulation (2.0 mM nitrate, 0.25 mM sulfur, 0.20 mM phosphorus, 0.62 mM potassium, 0.75 mM calcium, 0.17 mM magnesium, 0.25 mM ammonium, 20 μM iron, 9 μM manganese,0.3 μM cupper, 1.6 μM zinc, 20 μM boron, and 0.3 μM molybdenum), with an electrical conductivity (EC) and pH of 0.4 ± 0.1 dS m^−1^ and 6 ± 0.2, respectively. Each treatment was replicated three times, and the substrate trays were positioned randomly in the climate chamber. Daily rotation of the trays was performed to ensure homogenous light and humidity across the shelf surface. 

### 2.3. Growing Substrates Physicochemical Characterization

The physical properties of the five growing substrates, such as bulk density, total pore space, water holding capacity and air capacity, were appraised according to Di Gioia et al. [22]. For the assessment of chemical properties, substrate samples were dried to a constant weight at 105 °C. The EC and pH of the different substrates were determined in 1:2.5 substrate:water suspensions stirred overnight before measurements were performed. A benchtop electrical conductivity meter (SevenMulti, Metler-Toledo GmbH, Greifensee, Switzerland) and an electrochemical pH meter (SevenMulti; Mettler-Toledo GmbH, Schwerzenbach, Switzerland) were used for these measurements.

For the mineral content analysis of the substrates, 500 mg of each substate was suspended in 50 mL ultrapure water and shook in an orbital lab shaker (KS125 basic, IKA, Staufen, Germany) for 10 min. Later on, the water extracts were analyzed to determine the concentration of NO_3_^−^, P, K^+^, Ca^2+^, Mg^2+^, S, NH_4_^+^, Na^+^ and Cl^−^ by ion chromatography (ICS-3000, Dionex, Sunnyvale, CA, USA) with a conductivity detector. All physical and chemical analyzes were performed in triplicate, and the results are listed in Table 1.

### 2.4. Harvesting Schedule, Sampling, Growth Analysis and Colorimeter Measurement

Shortly before harvesting, the microgreens’ canopy color lightness (L*) was measured at six different points on each plastic tray using an 8 mm-aperture Minolta CR-400 Chroma Meter (Minolta Camera Co. Ltd., Osaka, Japan). All microgreens were harvested as soon as the second true leaf emerged, by cutting at the substrate level. The harvested material was weighed to determine the fresh weight, expressed in kg (fw) m^−2^. Directly after, 10 g of fresh microgreens were instantly stored in liquid nitrogen and then stored at −80 °C prior to liophilization in a freeze drier (Christ, Alpha 1-4, Osterode, Germany). 

Microgreens’ dry weight (dw) was measured on an analytical balance (XT120A; Precisa Gravimetrics, Dietikon, Switzerland) after desiccation of the remaining material in a forced-air oven at 65 °C until reaching constant weight. Dry matter (DM) content was also calculated and expressed as a percentage of microgreens’ fresh mass. The dry material (microgreens leaves and stems) was ground in a Wiley Mill to pass through an 841-microns screen and used for chemical analyses.

### 2.5. Mineral Analysis, Nitrate, Total Chlorophyll and Total Ascorbic Acid

Nitrate, macro-minerals and sodium concentrations were determined by ion chromatography (ICS-3000, Dionex, Sunnyvale, CA, USA) coupled to a conductivity detector, as described in Rouphael et al. [24]. The results were expressed in g kg^−1^ dw, except for nitrate that was converted to mg kg^−1^ fw based on each sample’s dw. Total chlorophyll content (mg kg^−1^ fw) was extracted by grinding 200 mg fw of microgreens in 80% ammoniacal acetone using a mortar and pestle; then the extract was centrifuged for 3 min at 3000g. The supernatant absorbance was read at 647 and 664 nm through a Hach DR 2000 spectrophotometer (Hach Co., Loveland, Colorado, USA) to determine the content of chlorophyll a and b, respectively, and their sum was taken as the total chlorophyll content [25].

As reported by Kampfenkel et al. [26], the sum of ascorbic and dehydroascorbic acids defined the total ascorbic acid concentration assessed by UV–Vis spectrophotometry (Hach DR 2000; Hach Co., Loveland, CO, USA). The quantification was carried out at 525 nm against an ascorbic acid (AA) standard calibration curve (5–100 μmol mL^−1^) and expressed in mg AA kg^−1^ fw.

### 2.6. Carotenoids and Polyphenols Extraction and Quantification

Lutein and β-carotene were extracted from lyophilized samples and separated on a Shimadzu HPLC Model LC 10 (Shimadzu, Osaka, Japan) using a reverse phase 250 × 4.6 mm, 5 μm Gemini C18 column (Phenomenex, Torrance, CA, USA) according to the method described by Kim et al. [27] and the modifications introduced by Kyriacou et al. [13]. Quantification was performed against calibration curves built with lutein and β-carotene external standards (5–100 μg mL^−1^) and results were expressed in mg kg^−1^ dw. The levels of carotenoids were calculated using the regression equation *y* = 1.163*x* − 994 (*r*^2^ = 0.992) for β-carotene and *y* = 1.053*x* − 0.651 (*r*^2^ = 0.990) for lutein. The limit of quantification (LOQ) was calculated for each standard and was determined as the lowest injected amount which could be reproducibly quantified (RSD ≤ 3%). The LOQ value was 0.25 ppm for both carotene and lutein. 

Polyphenols were also extracted from lyophilized samples and separated on a UHPLC system (UHPLC, Thermo Fisher Scientific, Waltham, MA, USA) equipped with a Kinetex 1.7 µm Biphenyl (100 × 2.1 mm) column (Phenomenex, Torrance, CA, USA) according to the conditions described by Llorach et al. [28] and Kyriacou et al. [13]. Mass spectrometry analysis was performed on a Q Exactive Orbitrap LC-MS/MS (Thermo Fisher Scientific, Waltham, MA, USA). Acquisition of polyphenolic compounds was carried out on parallel reaction monitoring (PRM). This modality of acquisition allows a targeted MS/MS analysis using the mass inclusion list and expected retention times of the target analytes, with a 30 s time window, with the Orbitrap spectrometer operating in negative mode at 17,500 FWHM (*m/z* 200). The AGC target was set to 2e5, with the maximum injection time of 20 ms. The precursor ions in the inclusion list were filtered by the quadrupole at an isolation window of *m/z* 2 and fragmented in an HCD collision cell set at 30 Kv. A mass tolerance of 5 ppm was employed. The instrument calibration was checked daily using a reference standard mixture obtained from Thermo Fisher Scientific.

### 2.7. Statistics

Experimental data were subjected to bifactorial (microgreens species × substrate) analysis of variance using SPSS 20 software package (IBM, Armonk, NY, USA). Treatment means were separated by Duncan’s Multiple Range Test performed at *p* ≤ 0.05. Yield and compositional characteristics of microgreens were subjected to principal component analysis (PCA) to explore relationships among variables and to compare the collective effects of substrates on these traits.

## 3. Results and Discussion

### 3.1. Substrate Physicochemical Properties

The five media presently evaluated as substrates for microgreens presented significant variation in physicochemical constitution (Table 1). The mechanical properties of a substrate, particularly its porosity, are associated with its bulk density (BD), which also reflects on the transport cost for its distribution [22]. The BD ranged from the lightest cellulose sponge (39.6 kg m^−3^) to the heaviest capillary mat (240.7 kg m^−3^), whereas peat moss, agave fiber, and coconut fiber had moderate BD (112.4–147.8 kg m^−3^). Nonetheless, all media registered a BD below 400 kg m^−3^, considered the maximum acceptable value according to the horticultural media inventory by Abad et al. [29]. Total pore space (TPS), which is the sum of a medium’s water-holding and air-holding capacities (WC and AC, respectively) was lowest in capillary mat (86.4% *v/v*) and highest in agave fiber (95.6 % *v/v*), yet all substrates were within the optimal TSP range (>85 %; [29]). However, substrates differed markedly in the type of porosity as indicated by differences in WC and AC (Table 1). Peat moss had the lowest WC (58.8 %) and inversely the highest AC (28.7 %), reflecting its low content of micropores and high content of macropores. The low AC of cellulose sponge, capillary mat and coconut fiber reflects their high relative content of micropores that provides a less favorable environment for root function. The above results carry important implications for the behavior of the tested substrates particularly with regard to the frequency and volume of irrigation. An ideal growing medium must combine physical properties sustaining a favorable balance between aeration and water holding during and between irrigation events so as to avoid water potential extremes and hypoxia conditions in the root zone [30]. The synthetic substrates of the capillary mat and cellulose sponge were restrained by low AC and their use for microgreen cultivation would require reduced frequency and/or volume of irrigation in order to sustain adequate root aeration [31]. Peat moss, on the other hand, showed higher AC but lower WC than the rest of the natural fiber substrates and would thus require more frequent controlled irrigation. It must be noted, however, that the generally optimal physical properties of peat may vary depending on the material used for its production, with less decomposed peats demonstrating higher WC than older and more decomposed material [32]. Further to peat, agave fiber combined near optimal physical properties of AC and WC, with the latter being only slightly supra-optimal. 

Peat moss and agave fiber also presented the lowest electrical conductivity (EC; 282 and 254 μS cm^−1^, respectively) whereas capillary mat had the highest (1258 mS cm^−1^; Table 1). The EC of coconut fiber (879 μS cm^−1^) was also notably high, derived likely from the processing of raw material with saline water in coastal areas of coconut production [30]. The variably high EC of coconut fiber draws additional cost for salt-leaching treatments of this otherwise cheap and renewable material, comparable to peat, before being used as a substrate for salt-sensitive crops like microgreens. Substrates of inherently high salt concentration have been shown to impair seed germination and seedling growth, although the severity of deleterious effects is species-dependent [33].

Coconut fiber, peat moss and capillary mat were all mildly acidic (pH 5.34–5.71) and their pH may be considered optimal for facilitating the availability of nutrients supplied through fertigation (Table 1; [29]). Agave fiber was the most acidic medium (pH 4.90) and cellulose sponge is outstandingly the most alkaline (pH 8.49). The pH of agave fiber may be easily adjusted through the nutrient solution, whereas cellulose sponge may require an acidification pretreatment. Residual nitrate was lowest in peat moss and capillary mat (0.24 and 0.54 g kg^−1^ dw, respectively) and highest in agave fiber (9.07 g kg^−1^ dw). Ammonium residue was highest in capillary mat (3.66 g kg^−1^ dw) and lowest in cellulose sponge and peat moss (0.20 and 0.42 g kg^−1^ dw). Cellulose sponge and capillary mat were deficient in phosphorous (0.01 and 0.07 g kg^−1^ dw, respectively), which was most abundant in coconut fiber (1.72 g kg^−1^ dw). Cellulose sponge was also very low in potassium content (1.93 g kg^−1^ dw), which was exceptionally high in coconut fiber (81.41 g kg^−1^ dw). Peat moss was the substrate highest in calcium content (30.34 g kg^−1^ dw) and capillary mat the lowest (3.34 g kg^−1^ sw). Relatively limited variation was observed for magnesium content which was higher in cellulose sponge (12.23 g kg^−1^ dw) than the rest media (2.66–5.73 g kg^−1^ dw). Sulphur was lowest in agave fiber (0.98 g kg^−1^ dw) and highest in capillary mat (9.15 g kg^−1^ dw). While the deficiencies in macronutrients described above may be remedied through the nutrient solution, the concentrations of sodium and chloride are critical and require close monitoring to avert salt stress on tender microgreens [34]. Peat moss was the substrate found to be lowest in both sodium (3.89 g kg^−1^ dw) and chloride (2.05 g kg^−1^ dw) content. Sodium content was highest in capillary mat (21.73 g kg^−1^ dw), while chloride was highest in coconut fiber (77.62 g kg^−1^ dw) and cellulose sponge (37.65 g kg^−1^ dw). Based on the overall physicochemical profile of the appraised substrates, peat moss and agave fiber present the most optimized environment for microgreens cultivation.

### 3.2. Fresh Biomass Yield, Dry Matter Content, Canopy Height and Color

**Figure 1:**
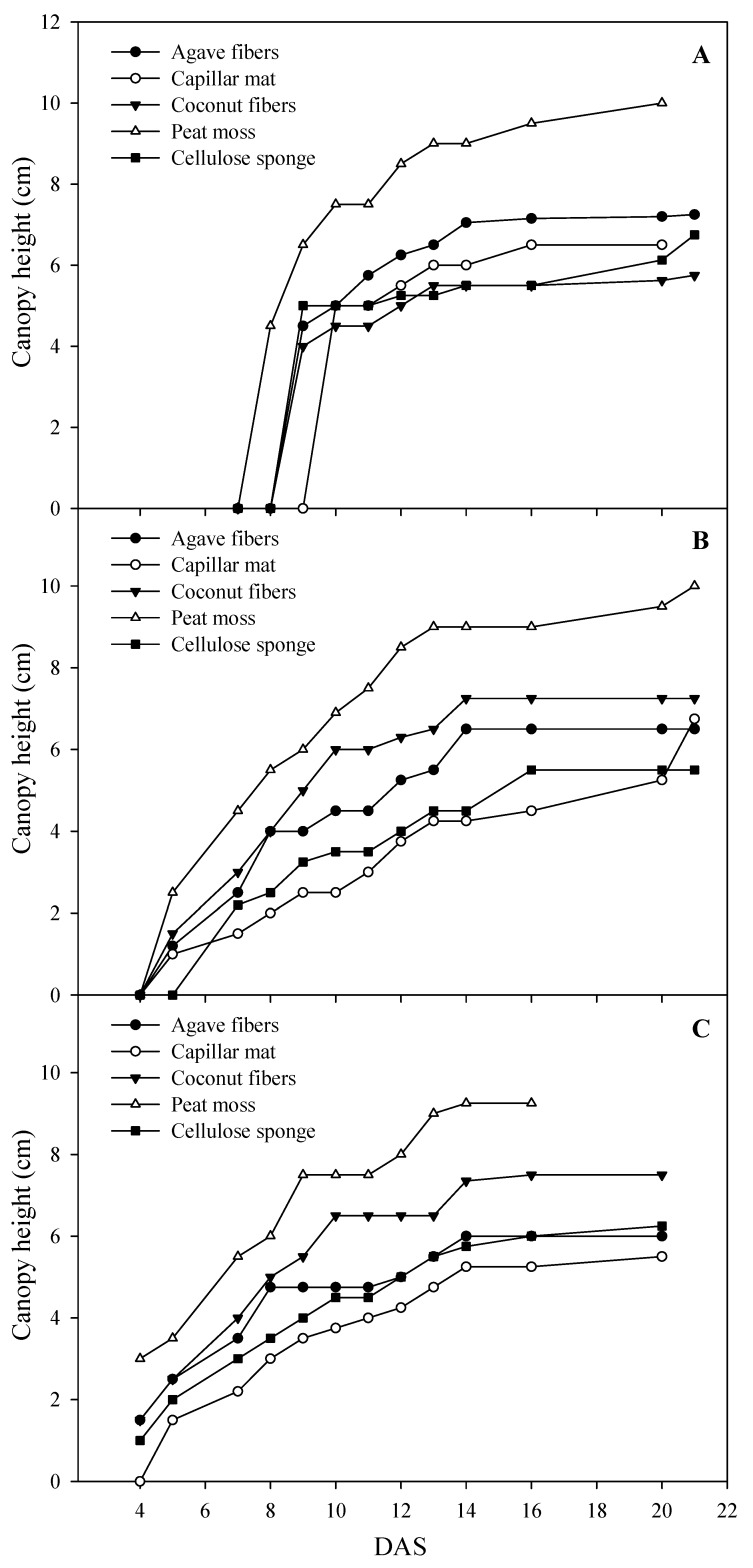
Canopy height of coriander (**A**), kohlrabi (**B**) and pak choi (**C**) microgreens while growing on natural fiber and synthetic substrates under controlled environment. DAS: days after sowing. All data are expressed as mean ± standard error, *n* = 3.

Species germination was progressively slower in the order pak choi, kohlrabi and coriander, as indicated by the intercepts and slopes of Figure 1A–C. Despite late germination, coriander microgreens exhibited faster growth than the other two species in the 2–4-day period immediately after emergence. The germination of coriander and pak choi was slowest on synthetic capillary mat, while for kohlrabi the slowest germination was on cellulose sponge. Considering the brief crop cycle of microgreens, the cost of controlled growth conditions and the demand for high turnover, a lag period of 1–2 days in the germination process may constitute an important setback associated with the above synthetic substrates [10]; which might in fact derive from their relatively low AC properties [31]. Moreover, all species demonstrated the tallest canopy throughout the growth period when grown on peat moss and the shortest when grown on capillary mat, except for coriander, which was shortest on coconut fiber (Figure 1A–C). Moreover, maximum canopy height was reached about two days earlier on peat moss compared to the rest of the substrates. It is thus apparent that the growth of all microgreen species availed of the optimal air-moisture-salinity conditions provided by peat moss and to a lesser extent by agave and coconut fibers. It is also evident that the inherently high EC of the synthetic substrates as well as that of coconut fiber can set back the growth of microgreens and prolong the crop cycle, especially of salt-sensitive species such as coriander [33,35].

The mean fresh yield obtained across species (1.66 ± 0.30 kg fw m^−2^) compared favorably to the yields reported for several microgreens’ species by previous workers [22,36]. Moreover, the current results indicate that the mean fresh yield of pak choi and kohlrabi microgreens was 28.4% higher than that of coriander, regardless of substrate (Table 2), which underscores previous reports of starkly differentiated growth rates and biomass production in different species of microgreens [10]. Regardless of interspecific differences in yield, microgreens of all species yielded outstandingly when grown on peat moss, amounting to a 55.1% increase compared to the other four substrates. Coconut fiber was the second-best substrate in terms of yield for kohlrabi and pak choi but not for coriander, reflecting the sensitivity of coriander to the high initial EC levels encountered in this medium that resulted in delayed germination and slower growth rate (Table 1; Figure 1; [33,35]). Dry yield was not differentiated among species; however, it was significantly affected by the choice of substrate (Table 2). It was highest on peat moss, which exceeded that of the other four substrates by 35.7%. However, aside from peat moss, which maximized the yield of all species, ranking the rest four substrates for dry yield was confounded by M × S interaction. In terms of dry matter content, coriander microgreens attained the highest mean value (13.6%), which was 23.4% higher than the mean value of kohlrabi and pak choi. Microgreens grown on peat moss and coconut fiber had the lowest dry matter content overall, which was 31.1% lower than the average of the other four substrates. Nevertheless, significant M × S interaction indicated that species response to substrate for dry matter content was not uniform. For instance, contrary to kohlrabi and pak choi, the dry matter content of coriander microgreens was highest when grown on coconut fiber, cellulose sponge and capillary mat (Table 2), all of which were noted for their high EC content (Table 1). Increase in dry matter content in response to salt stress has been previously reported for other salt-sensitive species such as lettuce [33,37]. Finally, canopy coloration, evaluated in terms of lightness (CIELAB parameter L*), incurred significant species and substrate effects without interaction. Microgreens canopy was progressively darker in the order coriander, kohlrabi, pak choi, expressed by significantly different and progressively lower L* values (Table 2). With respect to substrate, canopy color was significantly darker in microgreens grown on the synthetic substrates capillary mat and cellulose sponge compared to the natural fiber substrates of agave fiber, peat moss and coconut fiber. The lighter canopy color of microgreens grown on natural fibers is most probably linked to the faster growth and the generally lower dry matter content of microgreens grown on these media, which in turn is linked to their optimal aeration–water capacity balance [22,31,32]. Notwithstanding statistical differences, variation in canopy coloration was visually not readily perceptible, therefore it did not impact the marketable quality of microgreens.

### 3.3. Nitrate Concentration and Mineral Composition

Substrate effect on nitrate and mineral (P, K, Ca, Mg, S and Na) concentrations of microgreens differed across species as denoted by significant M × S interaction (Table 3). Overall, nitrate concentration was higher in the brassicaceous species pak choi (601.6 mg kg^−1^ fw) and kohlrabi (500.1 mg kg^−1^ fw) compared to coriander (398.5 mg kg^−1^ fw), which confirms that the general tendency of the *Brassicaceae* members for nitrate hyper-accumulation is as pertinent for microgreens as it is for their mature counterparts [38,39]. Significant differences in microgreens’ nitrate concentrations were also observed in response to substrate. In all species, the highest concentration was obtained on peat moss ($\bar{x}$ = 888.0 mg kg^−1^ fw), which was 54.6% higher than the mean concentration encountered in the rest substrates (403.1 mg kg^−1^ fw). Overall, the natural fiber substrates resulted in higher nitrate concentrations in microgreens compared to the synthetic substrates. Nitrate uptake is largely facilitated through the transpiration stream [39], therefore natural fibers abundant in macropores are expected to exert lower suction on water, which is readily available to facilitate higher transpiration and faster rates coupled with increased nitrate uptake [40]. The nitrate concentrations presently determined were substantially below the tolerance levels set for key salad crops (lettuce, spinach and rocket) by effective regulations [41]; moreover, the limited quantity of microgreens consumed ensures that the daily toxic threshold (3.7 mg kg^−1^ body weight) set by the World Health Organization and the European Union is not easily violated [42]. Nitrate intake from various dietary sources is nonetheless cumulative and the current study indicates that nitrate deprivation practices applied on microgreens grown on natural fiber substrates, peat moss in particular, should be examined in order to minimize consumer exposure to nitrate residues. Among others, such practices may include reducing the nitrate concentration of the nutrient solution and reverting to a nitrate-free solution for 2–5 days before harvest [2,39].

The role of macro-minerals in ameliorating nutritional deficiencies and maintaining homeostasis and metabolic functions in the human body has been well described [43]. The dietary contribution of the macro-minerals K, Mg, Na, P, and Ca from vegetal sources has been estimated at approximately 35%, 24%, 11%, 11% and 7%, respectively [44]. The relative abundance of macrominerals in the microgreen species presently examined was largely in accordance with previous reports covering microgreens of wide-ranging botanical taxa [13,14,22,45], which in order of decreasing concentrations was K > Ca > P>Mg > S > Na. This finding contributes towards establishing a reference base for the nutritional value of microgreens in terms of mineral content, for which there remains a paucity of information. Moreover, little is known on the impact that different types of substrate may have on microgreens’ mineral content. In the current study, natural fiber substrates including peat moss delivered microgreens of higher P concentration (4.26 ± 0.17 g kg^−1^ dw) than the synthetic substrates (3.83 ± 0.18 g kg^−1^ dw), notwithstanding significant M × S interaction (Table 3). Higher K content was obtained from microgreens grown on peat moss and coconut fiber; however, the synthetic capillary mat was also noted for delivering high K kohlrabi and pak choi microgreens. The highest Ca concentration in all species was clearly obtained in microgreens grown on peat moss, which was 54.2% higher than the mean concentration of the rest substrates. Peat moss also delivered coriander and kohlrabi microgreens of the highest Mg concentration, but substrate differences were more limited in the case of pak choi. Peat moss further sustained the highest S concentration in microgreens of all species. Finally, Na concentrations tended to be highest in microgreens grown on capillary mat and peat moss. In the former case, this stemmed from the high initial Na concentration of the capillary mat, whereas high evapotranspiration drove a buildup of Na in peat moss. Pronounced species differences in S concentration were observed, which was lower in coriander than kohlrabi and pak choi. High S content in the latter two species is unsurprising, since sulphur-rich glucosinolates constitute a signature trait of the *Brassicaceae* family [46]. Mg and P concentrations were the least variable across species. Of the three microgreens species examined, coriander was the species that accumulated the lowest concentrations of both nitrate and minerals overall.

### 3.4. Chlorophyll, Carotenoid and Ascorbate Content

The total chlorophyll content of microgreens varied more with respect to species (8.85–14.33 mg kg^−1^ fw) than substrate (11.16–13.13 mg kg^−1^ fw; Table 4). These levels were nearly two-fold higher than those found by Samuoliene et al. [16] in kohlrabi, mibuna and mustard microgreens. Other than varietal, this difference possibly reflects harvest performed at different developmental stages, i.e., at the cotyledonary stage, at the appearance of the first or second true leaf, the latter being the canonical stage for microgreens [10]. Kohlrabi had significantly lower total chlorophyll content that amounted to only 63.3% of the mean content found in coriander and pak choi microgreens. Higher chlorophyll content was obtained from microgreens grown on synthetic capillary mat and cellulose sponge than peat moss and coconut fiber, whereas intermediate levels were obtained on agave fiber. These substrate effects were similar to those manifested on canopy color and are likely associated with the higher growth rate and lower dry matter content of microgreens grown on natural fiber substrates which sustain optimal moisture–aeration conditions [22,31,32].

The content of microgreens in the major carotenoid compounds lutein and β-carotene contributes to their bioactive value, as both molecules are lipophilic antioxidants drawing light-absorbing and ROS-quenching properties from their long polyene chains [47]. Dietary supplementation with lutein has been associated with macular protection against oxidative damage and degeneration [48]; whereas β-carotene is a precursor of vitamin A, essential for growth, visual and immune functions. In the microgreen species presently examined, lutein content was highest in pak choi and lowest in kohlrabi (122.6 and 73.2 mg kg^−1^ fw, respectively; Table 4). Wide variation in lutein content was also observed between microgreens grown on cellulose sponge (70.4 mg kg^−1^ fw) and those grown on the rest four media ($\bar{x}$ = 105.1 mg kg^−1^ fw). Beta-carotene content was highest in coriander, followed by pak choi and then kohlrabi. Significant M × S interaction confounded the substrate effect. In the case of coriander, β-carotene was highest in microgreens grown on capillary mat; whereas, in kohlrabi and pak choi microgreens, differences between substrates were not statistically significant. Variability in the levels of lutein and β-carotene was higher in relation to species than in response to substrate.

Microgreens’ ascorbate content was overall highest in kohlrabi, followed by pak choi and lowest in coriander (Table 4). However, significant M × S interaction was observed as species responded differently to the five substrates examined. Ascorbate content in coriander microgreens was significantly lower on peat moss compared to the other media. In kohlrabi microgreens, ascorbate content was lower on peat moss and coconut fiber compared to agave fiber, capillary mat and cellulose sponge. Finally, cellulose sponge was the substrate that delivered the highest ascorbate content in pak choi microgreens, whereas the other four substrates had non-significant differences among them.

### 3.5. Phenolic Composition

Chromatographic separation and quantitation of phenolic compounds by Q Exactive Orbitrap LC-MS/MS enabled the assessment of twenty constituent polyphenolic profiles for the three species of microgreens evaluated (Table 5). The total sum of polyphenols was highest in kohlrabi (16.26 ± 0.78 mg g^−1^ dw) and lowest in pak choi (14.22 ± 0.78 mg g^−1^ dw), and it was overall higher than the range reported by Xiao et al. [5] (1.5–7.0 mg g^−1^ dw) and Bulgari et al. [36] (164–328 µg g^−1^ fw) through spectroscopic assessment of various species of microgreens. This might reflect variable developmental stages at harvest, genotypic differences and also differences in analytical methodology. Moreover, variation in the method of harvest applied and the severity of the mechanical trauma inflicted can affect phenolic accumulation via induced signaling that migrates to adjacent nonwounded tissue wherein it triggers respiratory climax and phenylpropanoid biosynthesis [49]. Comparatively wider variability in total polyphenols was encountered across substrate types than species of microgreens; significant interaction however highlighted a species-dependent response to substrate type (Table 5). Coriander and pak choi microgreens exhibited analogous responses as their phenolic content decreased on peat moss and coconut fiber. The same response was observed in Kohlrabi microgreens grown on coconut fiber, intriguingly though the highest phenolic content was obtained on peat moss. The response encountered in coriander and pak choi microgreens can be readily interpreted in the context of the faster growth rate and lack of physiological stress afforded by the optimal root environment in peat moss and coconut fiber substrates (Table 1; Figure 1A–C).

Of the twenty phenolic compounds quantitated, chlorogenic acid and rutin (quercetin-3-*O*-rutinoside) were the most abundant, accounting respectively for 48.5% and 35.5% of the total phenolic concentration (Table 5). The abundance of chlorogenic acid in young plant tissues and its decline at subsequent developmental stages has been demonstrated by Vallejo et al. [50] on *Brassica* seedlings, and probably relates to the intermediary role of chlorogenic acid in lignin biosynthesis [51]. Aside from this role, the abundance of chlorogenic acid in microgreens underpins their bioactive value based on chlorogenic acid’s antihypertensive effects on arterial pressure [52], and its putative anti-inflammatory action [53]. Previous work on eggplant has shown chlorogenic acid as the main contributor of in vitro antioxidant capacity, subject to genetic and environmental influence, such as developmental stage, genotype and cultural practices [54]. A recent study by Santos et al. [55] demonstrated that the inhibition of lipoperoxidation, A549 cell proliferation and antihypertensive activity were highest in plant extracts having chlorogenic acid as a major phenolic constituent. Similarly, the analgesic and anti-inflammatory activities of ethyl acetate extracts of *Kleinia pendula* (Forssk.) DC. were attributed to their phenolic acid content that was particularly rich in chlorogenic acid [56]. Santos et al. [55] also demonstrated that the ex vivo activity against low-density lipoprotein (LDL) oxidation, the protection of human erythrocytes and the cytotoxic-antiproliferative activity against HCT8 cancer cells was high in plant aqueous extracts rich in quercetin-3-rutinoside (rutin). In the present study, the mean phenolic content of microgreens across the species was made up of hydroxycinnamic acids and their derivatives by 49.8%, flavonol glycosides by 48.4% and flavone glycosides by 1.8%. This breakdown of polyphenolic constitution is relatively lower on hydroxycinnamic acids and higher on flavonol glycosides than previously found in cress, amaranth and mizuna microgreens [13,14], and reflects genotypic variation even within the *Brassicaceae* microgreens, as well as variation possibly introduced by slight differences in harvest maturity. It is in line, however, with the findings of previous researchers who demonstrated the predominance of quercetin and kampferol *O*-glycosides in the flavonol fraction of Brassica microgreens polyphenols [57,58] In this respect, the absence of kaempferol-3-*O*-(caffeoyl) sophoroside-7-*O*-glucoside and quercetin-3-*O*-glucuronide from coriander microgreens is unsurprising. Aside from the overall phenolic content, the presence of quercetin-3-*O*-glucuronide in the two brassicaceous microgreens (pak choi and kohlrabi) is important in view of studies demonstrating its protective role on dietary antioxidants found in the human plasma [59]. A recent study by Lesjak et al. [60] demonstrated that quercetin derivatives, such as quercetin-3-*O*- glucuronide, entering systemic circulation after their consumption may exert antioxidant and anti-inflammatory activity, thus highlighting the overall nutraceutical value of a quercetin-rich diet. Despite certain outstanding differences observed between species in the mean concentration of particular polyphenols (e.g., higher mean concentration of rutin in kohlrabi; of caffeic acid and kaempferol-3-*O*-rutinoside in coriander; of chlorogenic acid and kaempferol-3-*O*-(synapil)sophoroside-7-*O*-glucoside in pak choi), significant species–substrate interaction confounded putative species signature traits and complicated the interpretation of substrate effects with respect to particular phenolic constituents. Given the heightened respiratory and metabolic activity encountered in the rapidly growing and differentiating tissues of microgreens [10], even minimal differences in the stages of ontogeny at harvest may arrest disparate states of transient phenylpropanoid components thereby introducing qualitative variation in polyphenolic profiles, as previously demonstrated for mineral constituents during seedling ontogeny [7]. It is apparent nonetheless that substrates providing optimal environment for microgreens growth, such as peat moss, tend to enhance growth rate and biomass production at the expense of overall phenolic content. In this respect, the application of controlled stress (eustress) on microgreens growing on peat moss merits investigation as a means of enhancing phytochemical composition without significantly compromising crop performance and production turnover.

### 3.6. Principal Component Analysis of Nutritional and Functional Quality Parameters in Response to Substrate for Coriander, Kohlrabi and Pak Choi Microgreens

**Figure 2:**
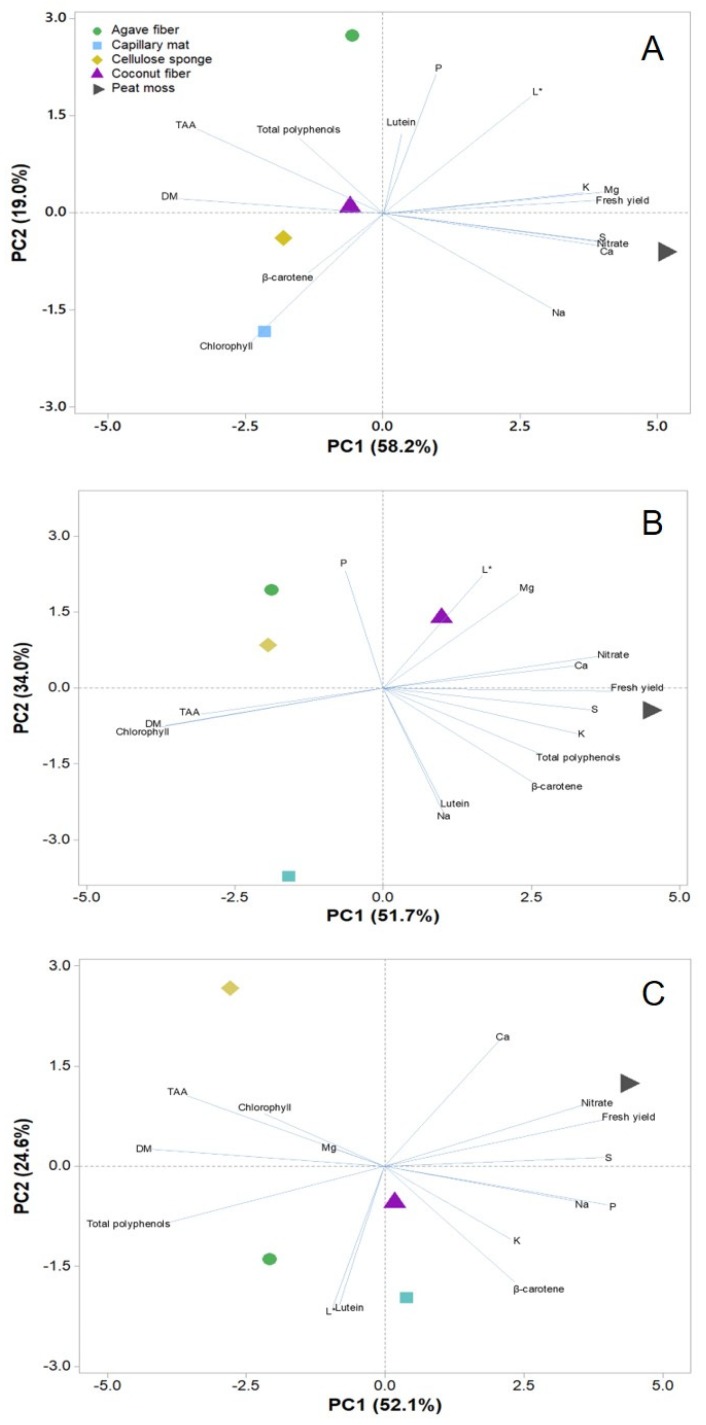
Principal component analysis loading plot of yield, mineral and phytochemical composition traits of coriander (**A**), kohlrabi (**B**) and pak choi (**C**) microgreens cultivated on natural fiber and synthetic substrates in a controlled growth environment.

Principal component analysis (PCA) has been previously demonstrated as an effective way of collectively representing sample population differences over multiple traits of productivity and quality in response to numerous cultivation factors [13,14,61]. In the current study, the PCA enabled a summarized view of the relations between microgreens crop performance and the compositional variables assessed (Figure 2A–C). Owing to the significant species-substrate interaction observed on most of these variables, PCA analysis was performed separately for each species. As a result, the species-dependent effects of the five substrates were better visualized and the quality of the PCA loading and score plots were improved, as indicated by the high percentage of the total variance (76.7–85.7%) accounted for the first two PCs. The main conclusive evidence provided with respect to the three species examined is the superiority of peat moss over other substrates in terms of microgreen yield and overall mineral content; however, peat moss also resulted in higher nitrate and lower dry matter content in microgreens across species. Chlorophyll content was particularly enhanced in microgreens grown on capillary mat and cellulose sponge. On the contrary, the concentrations of total polyphenols and major carotenoid molecules varied across substrates in a species-specific manner. For instance, total polyphenols were lowest on peat moss for coriander and pak choi microgreens, whereas kohlrabi microgreens grown on peat moss had the highest polyphenols. Therefore, the PCA representations underpin the main conclusion derived from the tabulated results (Table 2, Table 3, Table 4 and Table 5) that although the superiority of peat moss in several productive and compositional traits of microgreens is demonstrable, further research work is warranted to elucidate the interactive effects of substrate on certain quality traits of particular species. Although the synthetic substrates presently examined do represent competitive alternatives to peat moss, particularly in terms of growth rate, fresh yield and microgreens mineral composition, research work on finding viable alternatives to peat moss is warranted by sustainability issues associated with peat moss extraction [30], and also due to microbial safety aspects associated with the use of organic materials such as peat moss for microgreens production [22]. 

## 4. Conclusions

The current work constitutes a novel and unprecedented report on how the physicochemical properties of natural fiber and synthetic fiber substrates can influence the phytochemical content of microgreens. A key finding of the present work, which advances our understanding of the current and future literature on microgreens production and potential bioactive value, is that substrates which combine optimal physicochemical properties, such as peat moss, tend to promote faster growth and higher fresh yields that favor high production turnover; however, this is achieved at the expense of reduced phytochemical content, the foremost of polyphenols. Therefore, controlled stress applications (e.g., osmotic stress) on microgreens growing on such media warrants investigation as a means of enhancing phytochemical composition without substantial compromise in crop performance and production turnover. Substrates promoting fast growth (e.g., peat moss) also tend to promote nitrate accumulation in microgreens, especially in brassicaceous ones that are known nitrate hyperaccumulators. Therefore, nitrate deprivation practices should be considered for microgreens grown on such substrates in order to minimize consumer exposure to nitrates.

## Figures and Tables

**Table 1:** Physicochemical characterization of natural fiber and synthetic substrates used for coriander, kohlrabi and pak choi microgreens production in a controlled environment. All data are expressed as mean ± standard error, *n* = 3.

Physicochemical Parameters	Substrate					Significance
	Agave Fiber	Capillary Mat	Cellulose Sponge	Coconut Fiber	Peat Moss	
Bulk density (kg m^−3^)	144.2 ± 4.49^b^	240.7 ± 6.24^a^	39.6 ± 0.23^d^	112.4 ± 0.19^c^	147.8 ± 1.15^b^	***
Total pore space (% *v/v*)	95.6 ± 0.28^a^	86.4 ± 0.52^d^	93.8 ± 0.42^b^	90.8 ± 0.16^c^	87.4 ± 0.16^d^	***
Water-holding capacity (% *v/v*)	76.6 ± 0.3^b^	71.1 ± 0.5^d^	80.2 ± 0.58^a^	74 ± 0.12^c^	58.8 ± 0.27^e^	***
Air capacity (% *v/v*)	19 ± 0.25^b^	15.3 ± 0.21^d^	13.7 ± 0.61^e^	16.8 ± 0.07^c^	28.7 ± 0.27^a^	***
EC (μS cm^−1^)	254 ± 0.50^e^	1258 ± 8.00^a^	702 ± 0.50^c^	879 ± 1.00^b^	282 ± 1.50^d^	***
pH	4.90 ± 0.005^e^	5.71 ± 0.010^b^	8.49 ± 0.010^a^	5.34 ± 0.005^d^	5.48 ± 0.005^c^	***
NO_3_ (g kg^−1^ dw)	9.07 ± 0.21^a^	0.54 ± 0.11^d^	1.16 ± 0.06^c^	2.63 ± 0.18^b^	0.24 ± 0.05^d^	***
P (g kg^−1^ dw)	0.67 ± 0.03^c^	0.07 ± 0.00^d^	0.01 ± 0.00^e^	1.72 ± 0.00^a^	1.04 ± 0.01^b^	***
K (g kg^−1^ dw)	15.00 ± 0.32^b^	13.39 ± 0.03^bc^	1.93 ± 0.92^d^	81.41 ± 1.83^a^	12.10 ± 0.88^c^	***
Ca (g kg^−1^ dw)	10.66 ± 0.02^c^	3.44 ± 0.26^e^	9.89 ± 0.09^d^	16.67 ± 0.29^b^	30.34 ± 0.19^a^	***
Mg (g kg^−1^ dw)	4.92 ± 0.95^b^	2.75 ± 0.74^b^	12.23 ± 0.59^a^	5.73 ± 0.33^b^	2.66 ± 1.17^b^	**
S (g kg^−1^ dw)	0.98 ± 0.14^d^	9.15 ± 0.00^a^	1.72 ± 0.01^c^	1.10 ± 0.16^d^	6.09 ± 0.09^b^	***
NH_4_ (g kg^−1^ dw)	2.12 ± 0.06^b^	3.66 ± 0.05^a^	0.20 ± 0.16^c^	1.68 ± 0.05^b^	0.42 ± 0.16^c^	***
Na (g kg^−1^ dw)	6.72 ± 0.08^d^	21.73 ± 0.16^a^	12.18 ± 0.37^c^	19.80 ± 0.11^b^	3.89 ± 0.29^e^	***
Cl (g kg^−1^ dw)	9.58 ± 0.05^c^	6.42 ± 0.77^d^	37.65 ± 0.40^b^	77.52 ± 1.09^a^	2.05 ± 0.06^e^	***

ns, **, *** Nonsignificant or significant at *p* ≤ 0.01, and 0.001, respectively. Different superscript letters (a–e) within each row indicate significant differences according to Duncan’s multiple-range test (*p* = 0.05).

**Table 2:** Fresh and dry yield, dry matter content and canopy lightness (L*) of coriander, kohlrabi and pak choi substrates grown on natural fiber and synthetic substrates under a controlled environment. All data are expressed as mean ± standard error, *n* = 3.

Source of Variance	Yield (kg fw m^−^^2^)	Dry Weight (g m^−2^)	Dry Matter (%)	L*
Microgreens Species (M)				
Coriander	1.31 ± 0.14^b^	169.03 ± 11.06	13.59 ± 0.52^a^	33.68 ± 0.30^a^
Kohlrabi	1.80 ± 0.24^a^	182.89 ± 17.63	10.71 ± 0.37^b^	28.52 ± 0.26^b^
Pak choi	1.86 ± 0.20^a^	172.75 ± 8.18	10.12 ± 0.64^b^	23.87 ± 0.27^c^
Substrate (S)				
Agave fiber	1.18 ± 0.05^c^	143.46 ± 8.92^c^	12.16 ± 0.42^b^	29.57 ± 1.39^a^
Capillary mat	1.18 ± 0.12^c^	134.73 ± 7.17^c^	11.95 ± 0.68^b^	27.98 ± 1.26^c^
Cellulose sponge	1.31 ± 0.06^c^	172.81 ± 5.22^b^	13.30 ± 0.52^a^	28.27 ± 1.57^bc^
Coconut fiber	1.65 ± 0.18^b^	178.71 ± 9.43^b^	11.54 ± 0.94^b^	28.72 ± 1.36^abc^
Peat moss	2.96 ± 0.19^a^	244.75 ± 17.17^a^	8.43 ± 0.57^c^	28.91 ± 1.64^ab^
M × S				
Coriander × Agave fiber	1.28 ± 0.04^ef^	175.33 ± 8.20^cd^	13.66 ± 0.39^b^	34.49 ± 0.19
Coriander × Capillary mat	0.80 ± 0.08^g^	111.04 ± 8.59^g^	13.91 ± 0.39^ab^	32.55 ± 0.90
Coriander × Cellulose sponge	1.16 ± 0.12^efg^	174.67 ± 14.95^cd^	15.11 ± 0.32^a^	33.63 ± 0.49
Coriander × Coconut fiber	1.02 ± 0.02^fg^	153.66 ± 5.15^def^	15.12 ± 0.74^a^	33.27 ± 0.05
Coriander × Peat moss	2.27 ± 0.10^b^	230.45 ± 13.00^b^	10.12 ± 0.14^ef^	34.49 ± 0.82
Kohlrabi × Agave fiber	1.08 ± 0.04^fg^	121.70 ± 4.95^fg^	11.29 ± 0.12^de^	29.16 ± 0.48
Kohlrabi × Capillary mat	1.20 ± 0.02^ef^	144.69 ± 8.70^def^	12.07 ± 0.85^cd^	27.21 ± 0.31
Kohlrabi × Cellulose sponge	1.41 ± 0.03^ef^	164.00 ± 3.24^de^	11.65 ± 0.10^d^	28.32 ± 0.49
Kohlrabi × Coconut fiber	1.86 ± 0.23^cd^	180.27 ± 20.61^cd^	9.72 ± 0.42^f^	28.93 ± 0.59
Kohlrabi × Peat moss	3.43 ± 0.18^a^	303.77 ± 15.78^a^	8.84 ± 0.05^f^	28.98 ± 0.47
Pak choi × Agave fiber	1.17 ± 0.13^efg^	133.33 ± 8.20^efg^	11.54 ± 0.50^d^	25.05 ± 0.68
Pak choi × Capillary mat	1.53 ± 0.17^de^	148.44 ± 6.60^def^	9.86 ± 0.76^f^	24.19 ± 0.46
Pak choi × Cellulose sponge	1.37 ± 0.08^ef^	179.77 ± 5.31^cd^	13.14 ± 0.35^bc^	22.85 ± 0.39
Pak choi × Coconut fiber	2.08 ± 0.13^bc^	202.19 ± 5.12^bc^	9.76 ± 0.43^f^	23.97 ± 0.49
Pak choi × Peat moss	3.16 ± 0.14^a^	200.02 ± 16.64^bc^	6.31 ± 0.32^g^	23.27 ± 0.11
Significance				
Microgreens species (M)	***	ns	***	***
Substrate (S)	***	***	***	**
M × S	***	***	***	ns

ns, **, *** Nonsignificant or significant at *p* ≤ 0.01, and 0.001, respectively. Different superscript letters (a–g) within each column indicate significant differences according to Duncan’s multiple-range test (*p* = 0.05).

**Table 3:** Nitrate and mineral content of coriander, kohlrabi and pak choi substrates grown on natural fiber and synthetic substrates under a controlled environment. All data are expressed as mean ± standard error, *n* = 3.

Source of Variance	Nitrate (mg kg^−1^ fw)	P (g kg^−1^ dw)	K (g kg^−1^ dw)	Ca (g kg^−1^ dw)	Mg (g kg^−1^ dw)	S (g kg^−1^ dw)	Na (g kg^−1^ dw)
Microgreens species (M)							
Coriander	398.5 ± 80.0^c^	4.14 ± 0.25^b^	15.60 ± 0.77^b^	6.46 ± 1.26^c^	4.27 ± 0.21	0.98 ± 0.17^c^	0.48 ± 0.10^c^
Kohlrabi	500.1 ± 59.6^b^	3.93 ± 0.18^b^	10.88 ± 0.69^c^	12.98 ± 1.21^a^	4.25 ± 0.21	3.10 ± 0.22^b^	1.78 ± 0.37^b^
Pak choi	601.6 ± 52.9^a^	4.92 ± 0.29^a^	21.20 ± 1.31^a^	9.58 ± 0.73^b^	4.22 ± 0.11	4.03 ± 0.52^a^	2.68 ± 0.47^a^
Substrate (S)							
Agave fiber	361.3 ± 48.5^c^	4.68 ± 0.32^a^	13.69 ± 1.52^c^	8.04 ± 1.15^c^	4.51 ± 0.19^b^	2.23 ± 0.37^bc^	0.44 ± 0.09^d^
Capillary mat	324.3 ± 52.7^c^	3.95 ± 0.38^bc^	16.32 ± 1.94^b^	6.12 ± 0.71^d^	3.45 ± 0.16^d^	2.64 ± 0.52^b^	3.16 ± 0.66^a^
Cellulose sponge	396.1 ± 60.7^c^	3.70 ± 0.27^c^	11.97 ± 1.29^c^	9.43 ± 1.29^b^	3.93 ± 0.14^c^	1.82 ± 0.36^c^	0.64 ± 0.14^d^
Coconut fiber	530.5 ± 71.3^b^	4.82 ± 0.20^a^	19.06 ± 2.21^a^	7.70 ± 0.89^c^	4.42 ± 0.08^b^	2.07 ± 0.38^c^	1.25 ± 0.28^c^
Peat moss	888.0 ± 31.8^a^	4.49 ± 0.41^ab^	18.43 ± 1.41^a^	17.07 ± 1.16^a^	4.92 ± 0.22^a^	4.77 ± 0.80^a^	2.75 ± 0.53^b^
M × S							
Coriander × Agave fiber	236.8 ± 25.2^ef^	5.17 ± 0.92^ab^	15.62 ± 1.50^efg^	3.94 ± 0.29^h^	4.26 ± 0.49^cde^	0.80 ± 0.10^d^	0.17 ± 0.01^g^
Coriander × Capillary mat	186.5 ± 43.2^f^	3.53 ± 0.22^cd^	13.82 ± 1.07^efg^	3.58 ± 0.42^h^	3.61 ± 0.12^f^	0.81 ± 0.09^d^	0.69 ± 0.03^def^
Coriander × Cellulose sponge	255.4 ± 15.9^ef^	3.20 ± 0.06^cd^	12.18 ± 0.19^gh^	4.39 ± 0.10^h^	3.70 ± 0.09^ef^	0.48 ± 0.05^d^	0.14 ± 0.00^g^
Coriander × Coconut fiber	333.1 ± 49.0^def^	4.65 ± 0.24^abc^	16.77 ± 1.03^def^	4.58 ± 0.31^h^	4.27 ± 0.17^cde^	0.62 ± 0.04^d^	0.33 ± 0.02^fg^
Coriander × Peat moss	980.6 ± 37.2^a^	4.15 ± 0.11^bcd^	19.61 ± 0.60^cd^	15.80 ± 0.68^b^	5.53 ± 0.19^a^	2.19 ± 0.09^c^	1.09 ± 0.04^de^
Kohlrabi × Agave fiber	301.3 ± 6.3^ef^	4.53 ± 0.20^abc^	8.03 ± 0.26i	11.79 ± 0.25^de^	4.87 ± 0.16^abc^	2.71 ± 0.08^c^	0.36 ± 0.02efg
Kohlrabi × Capillary mat	276.4 ± 4.6^ef^	3.06 ± 0.13^d^	11.54 ± 0.48^hi^	8.31 ± 0.30^f^	2.89 ± 0.08^g^	2.79 ± 0.11^c^	4.01 ± 0.34^b^
Kohlrabi × Cellulose sponge	399.8 ± 15.6^cde^	3.95 ± 0.30^bcd^	7.99 ± 0.36^i^	12.84 ± 0.21^cd^	3.90 ± 0.05^def^	2.67 ± 0.19^c^	0.71 ± 0.10^defg^
Kohlrabi × Coconut fiber	729.0 ± 64.9^b^	4.64 ± 0.10^abc^	13.07 ± 0.83^fgh^	10.60 ± 0.23^e^	4.49 ± 0.11^bcd^	2.63 ± 0.20^c^	1.21 ± 0.14^d^
Kohlrabi × Peat moss	793.8 ± 24.8^ab^	3.46 ± 0.12^cd^	13.76 ± 0.56^efg^	21.35 ± 1.12^a^	5.08 ± 0.07^ab^	4.71 ± 0.09^b^	2.59 ± 0.06^c^
Pak choi × Agave fiber	545.8 ± 31.6^c^	4.32 ± 0.36^bcd^	17.43 ± 0.64^de^	8.37 ± 0.45^f^	4.39 ± 0.21^cde^	3.17 ± 0.26^c^	0.78 ± 0.05^defg^
Pak choi × Capillary mat	509.9 ± 59.6^cd^	5.28 ± 0.52^ab^	23.59 ± 1.70^b^	6.49 ± 0.11^g^	3.83 ± 0.22^def^	4.31 ± 0.34^b^	4.79 ± 0.55^a^
Pak choi × Cellulose sponge	533.0 ± 156.4^c^	3.96 ± 0.77^bcd^	15.74 ± 2.19^efg^	11.06 ± 0.15^e^	4.19 ± 0.39^cde^	2.32 ± 0.36^c^	1.07 ± 0.08^def^
Pak choi × Coconut fiber	529.5 ± 123.2^c^	5.19 ± 0.54^ab^	27.33 ± 1.40^a^	7.92 ± 0.56^f^	4.52 ± 0.14^bcd^	2.95 ± 0.24^c^	2.22 ± 0.21^c^
Pak choi × Peat moss	889.6 ± 37.4^ab^	5.85 ± 0.69^a^	21.91 ± 2.39^bc^	14.08 ± 0.15^c^	4.16 ± 0.24^cde^	7.41 ± 0.93^a^	4.56 ± 0.54^ab^
Significance							
Microgreens species (M)	***	***	***	***	ns	***	***
Substrate (S)	***	**	***	***	***	***	***
M × S	***	**	**	***	***	***	***

ns, **, *** Nonsignificant or significant at *p* ≤ 0.01, and 0.001, respectively. Different superscript letters (a–i) within each column indicate significant differences according to Duncan’s multiple-range test (*p* = 0.05).

**Table 4:** Chlorophyll, lutein, β-carotene and ascorbate content of coriander, kohlrabi and pak choi substrates grown on natural fiber and synthetic substrates under a controlled environment. All data are expressed as mean ± standard error, *n* = 3.

Source of Variance	Total Chlorophyll (mg kg^−1^ fw)	Lutein (mg kg^−1^ dw)	β-Carotene (mg kg^−1^ dw)	Total Ascorbic Acid (mg ascorbic acid kg^−1^ dw)
Microgreens species (M)				
Coriander	13.63 ± 0.38^a^	98.6 ± 8.8^b^	325.1 ± 38.1^a^	121.40 ± 5.77^c^
Kohlrabi	8.85 ± 0.37^b^	73.2 ± 5.9^c^	183.1 ± 15.5^c^	199.87 ± 8.77^a^
Pak choi	14.33 ± 0.59^a^	122.6 ± 7.5^a^	236.8 ± 19.0^b^	177.12 ± 5.38^b^
Substrate (S)				
Agave fiber	12.59 ± 0.90^ab^	109.5 ± 15.7^a^	233.2 ± 31.6^b^	179.79 ± 12.16^a^
Capillary mat	13.13 ± 0.89^a^	112.3 ± 9.8^a^	351.1 ± 56.0^a^	173.37 ± 13.46^a^
Cellulose sponge	13.07 ± 1.17^a^	70.4 ± 8.2^b^	153.8 ± 11.0^c^	187.39 ± 16.08^a^
Coconut fiber	11.16 ± 0.89^b^	103.3 ± 8.6^a^	250.3 ± 28.9^b^	150.35 ± 9.77^b^
Peat moss	11.39 ± 1.17^b^	95.1 ± 11.0^a^	253.2 ± 23.9^b^	139.75 ± 14.03^b^
M × S				
Coriander × Agave fiber	12.70 ± 1.04	121.0 ± 23.5	312.9 ± 36.0^bc^	146.16 ± 3.23^bcd^
Coriander × Capillary mat	15.03 ± 0.84	99.4 ± 15.8	533.3 ± 103.1^a^	128.92 ± 4.93^cd^
Coriander × Cellulose sponge	13.76 ± 0.24	57.8 ± 7.2	169.1 ± 18.6^def^	128.01 ± 6.92^cd^
Coriander × Coconut fiber	13.78 ± 0.18	120.4 ± 13.2	355.2 ± 25.4^b^	116.41 ± 8.80^d^
Coriander × Peat moss	12.89 ± 1.22	94.3 ± 19.3	255.0 ± 33.3^bcd^	87.52 ± 7.88^e^
Kohlrabi × Agave fiber	9.68 ± 0.32	58.0 ± 7.3	134.9 ± 16.7^ef^	219.14 ± 19.37^a^
Kohlrabi × Capillary mat	10.03 ± 0.99	101.9 ± 16.5	234.5 ± 29.3^bcd^	217.77 ± 13.18^a^
Kohlrabi × Cellulose sponge	9.35 ± 0.30	52.2 ± 4.2	122.6 ± 15.9^f^	226.75 ± 11.88^a^
Kohlrabi × Coconut fiber	7.85 ± 0.23	73.8 ± 6.5	180.8 ± 18.6^cde^	158.42 ± 11.07^bc^
Kohlrabi × Peat moss	7.34 ± 0.92	79.8 ± 8.2	242.7 ± 22.9^bcd^	177.27 ± 7.44b
Pak choi × Agave fiber	15.40 ± 0.50	149.4 ± 13.3	252.0 ± 46.8^bcd^	174.08 ± 5.81^b^
Pak choi × Capillary mat	14.34 ± 0.77	135.5 ± 14.8	285.6 ± 24.9^bcd^	173.42 ± 1.85^b^
Pak choi × Cellulose sponge	16.09 ± 2.10	101.2 ± 3.6	169.6 ± 10.9^def^	207.41 ± 13.28^a^
Pak choi × Coconut fiber	11.86 ± 0.50	115.7 ± 4.7	214.9 ± 22.1^cde^	176.22 ± 1.28^b^
Pak choi × Peat moss	13.94 ± 1.19	111.2 ± 27.8	261.9 ± 71.7^bcd^	154.45 ± 8.26^bc^
Significance				
Microgreens species (M)	***	***	***	***
Substrate (S)	*	**	***	***
M × S	ns	ns	*	*

ns, *, **, *** Nonsignificant or significant at *p* ≤ 0.05, 0.01, and 0.001, respectively. Different superscript letters (a–f) within each column indicate significant differences according to Duncan’s multiple-range test (*p* = 0.05).

**Table 5:** Phenolic composition of coriander, kohlrabi and pak choi substrates grown on natural fiber and synthetic substrates under a controlled environment. All data are expressed as mean ± standard error, *n* = 3.

Source of variance	Caffeic acid	Caffeic acid hexoside	Chlorogenic acid	*p*-Coumaroylquinic acid	Dicaffeoylquinic acid	Ferulic acid	Feruloyl quinic acid	Feruloylglycoside	Kaempferol-3-O-(caffeoyl) sophoroside-7-O-glucoside	Kaempferol-3-O-(feruloyl) sophoroside-7-O-glucoside	Kaempferol-3-O-(synapil) sophoroside-7-O-glucoside	Kaempferol-3-O-rutinoside	Luteolina-3-O-rutinoside	Quercetin sophoroside	Quercetin-3-O-(feruloyl) sophoroside-7-O-glucoside	Quercetin-3-O-sophoroside-7-O-glucoside	Quercetina-3-O-glucoside	Quercetina-3-O-glucuronide	Rosmarinic acid	Rutin	Total polyphenols
	(µg g^−1^ dw)	(µg g^−1^ dw)	(µg g^−1^ dw)	(µg g^−1^ dw)	(µg g^−1^ dw)	(µg g^−1^ dw)	(µg g^−1^ dw)	(µg g^−1^ dw)	(µg g^−1^ dw)	(µg g^−1^ dw)	(µg g^−1^ dw)	(µg g^−1^ dw)	(µg g^−1^ dw)	(µg g^−1^ dw)	(µg g^−1^ dw)	(µg g^−1^ dw)	(µg g^−1^ dw)	(µg g^−1^ dw)	(µg g^−1^ dw)	(µg g^−1^ dw)	(mg g^−1^ dw)
Microgreens species (M)																					
Coriander	125.84 ± 16.7^a^	88.53 ± 4.1^a^	6367 ± 549^b^	64.85 ± 2.11^a^	33.52 ± 1.22^a^	20.55 ± 0.04^a^	533.8 ± 20.6^a^	93.14 ± 2.6^c^	nd	319.0 ± 44.7^a^	86.9 ± 14^c^	566.5 ± 51^a^	563 ± 51.3^a^	83.99 ± 6.0^a^	nd	4.89 ± 0.08^c^	381.4 ± 23.9^a^	791.1 ± 48^a^	20.41 ± 0.08^c^	5109 ± 367^b^	15.25 ± 0.57^ab^
Kohlrabi	40.34 ± 4.0^b^	38.24 ± 4.5^b^	6776 ± 516^b^	22.99 ± 0.20^c^	19.72 ± 0.03^b^	19.55 ± 0.01^c^	78.95 ± 3.3^c^	154.79 ± 17.0^b^	25.47 ± 1.44 ^b^	169.9 ± 6.3^b^	291.7 ± 10^b^	114.1 ± 3^b^	164 ± 21.0^b^	4.40 ± 0.1^c^	10.79 ± 0.40^b^	30.45 ± 1.08^b^	222.0 ± 8.3^b^	nd	20.64 ± 0.03^b^	8052 ± 735^a^	16.26 ± 0.78^a^
Pak choi	32.71 ± 1.1^c^	38.69 ± 3.1^b^	9026 ± 837^a^	25.90 ± 0.62^b^	19.59 ± 0.03^b^	20.24 ± 0.03^b^	117.66 ± 8.4^b^	314.81 ± 50.7^a^	35.56 ± 2.25^a^	113.4 ± 8.4^c^	862.5 ± 40^a^	128.4 ± 5^b^	146 ± 8.3^b^	10.80 ± 0.3^b^	45.50 ± 2.93^a^	71.93 ± 4.02^a^	95.4 ± 6.0^c^	nd	20.78 ± 0.05^a^	3096 ± 365^c^	14.22 ± 0.78^b^
Substrate (S)																					
Agave fiber	48.79 ± 7.2^d^	55.07 ± 8.1^b^	9947 ± 1095^a^	40.40 ± 4.78^ab^	23.75 ± 1.39^b^	20.12 ± 0.11^b^	281.26 ± 54.2^a^	84.61 ± 4.6^d^	16.68 ± 0.89^c^	136.4 ± 5.3^c^	363.4 ± 70^c^	265.3 ± 43^a^	238 ± 47.6^c^	43.05 ± 12.6^a^	18.55 ± 3.14e	21.99 ± 3.37^c^	216.3 ± 40.7^bc^	754.1 ± 46^bc^	20.52 ± 0.07^b^	4027 ± 581^d^	16.11 ± 0.71^a^
Capillary mat	56.56 ± 4.7^c^	49.05 ± 5.9^bc^	8625 ± 780^b^	42.01 ± 5.66^a^	22.98 ± 1.14^b^	20.08 ± 0.10b	260.1 ± 48.2^ab^	107.30 ± 9.6^c^	32.90 ± 1.03^b^	180.8 ± 18.1^b^	512.5 ± 125^a^	321.6 ± 80^a^	340 ± 76.5^ab^	26.88 ± 6.8^b^	35.98 ± 6.7^b^	33.97 ± 6.30^b^	239.2 ± 33.1^ab^	1057.9 ± 90^a^	20.46 ± 0.07^b^	5061 ± 390^bc^	16.32 ± 0.39^a^
Cellulose sponge	119.46 ± 31.6^a^	49.87 ± 6.9^bc^	8757 ± 579^b^	32.47 ± 3.17^d^	24.74 ± 1.70^b^	20.05 ± 0.09^b^	210.8 ± 42.2^c^	272.90 ± 53.0^b^	34.61 ± 1.06^ab^	143.1 ± 13.6^c^	446.2 ± 85^b^	156.9 ± 20^b^	223 ± 12.9^c^	44.30 ± 13.1^a^	22.35 ± 3.53^d^	35.45 ± 6.10b	274.8 ± 35.4^a^	580.6 ± 66^c^	20.61 ± 0.05^b^	4584 ± 586^cd^	15.65 ± 0.44^a^
Coconut fiber	40.81 ± 7.1^e^	43.28 ± 9.6^c^	5290 ± 466^c^	37.85 ± 5.06^bc^	26.98 ± 2.94^a^	20.22 ± 0.13^a^	229.8 ± 60.8^bc^	94.75 ± 3.4^d^	30.94 ± 3.59^b^	363.1 ± 71.1^a^	362.0 ± 63^c^	304.8 ± 73^a^	260 ± 79.0^bc^	28.56 ± 7.3^b^	39.51 ± 9.02^a^	43.40 ± 9.53^a^	171.1 ± 17.1^c^	617.5 ± 98^c^	20.95 ± 0.09^a^	5646 ± 543^b^	13.24 ± 0.75^b^
Peat moss	65.86 ± 4.4^b^	78.49 ± 3.2^a^	4331 ± 500^d^	36.84 ± 5.21^c^	22.95 ± 1.18^b^	20.11 ± 0.11^b^	235.5 ± 54.7^bc^	378.32 ± 60.8^a^	37.45 ± 4.35^a^	180.3 ± 23.7 ^b^	384.5 ± 66^c^	299.7 ± 74^a^	394 ± 63.3^a^	22.53 ± 5.7^b^	24.34 ± 3.96^c^	43.97 ± 8.71^a^	263.4 ± 35.4^ab^	945.6 ± 84^ab^	20.51 ± 0.07^b^	7776 ± 1377^a^	14.91 ± 1.68^a^
M × S																					
Coriander × Agave fiber	86.11 ± 9.7^b^	100.90 ± 5.7^a^	8244 ± 1149^de^	67.41 ± 2.82^b^	31.82 ± 0.32^bc^	20.53 ± 0.08^bc^	587.9 ± 25.4^a^	86.11 ± 2.4^efg^	nd	116.2 ± 2.7^f^	99.8 ± 15^g^	505.2 ± 40^b^	502 ± 39.8^bc^	114.72 ± 7.9^a^	nd	5.11 ± 0.17^h^	430.1 ± 49.5a	754.1 ± 46b	20.18 ± 0.04^h^	5169 ± 344^cde^	16.94 ± 1.35^bcd^
Coriander × Capillary mat	78.25 ± 0.5^bc^	82.48 ± 2.6^a^	4807 ± 762^gh^	73.92 ± 4.53^a^	29.45 ± 0.88^c^	20.38 ± 0.10^cd^	537.0 ± 19.9^a^	84.89 ± 5.2^fg^	nd	283.6 ± 1.9^b^	55.0 ± 14^g^	741.7 ± 108^a^	738 ± 108.4^a^	64.98 ± 5.4^bc^	nd	4.80 ± 0.17^h^	378.8 ± 42.1^a^	1057.9 ± 90^a^	20.18 ± 0.07^h^	6741 ± 813^bc^	15.80 ± 0.80^bcdef^
Coriander x Cellulose sponge	303.39 ± 3.0^a^	83.80 ± 9.9^a^	9850 ± 679^cd^	49.45 ± 3.88^c^	34.60 ± 0.61^b^	20.46 ± 0.04^cd^	434.1 ± 53.1^b^	82.59 ± 1.6f^g^	nd	137.8 ± 2.7^ef^	94.5 ± 26^g^	250.4 ± 37^c^	243 ± 37.3^de^	117.17 ± 12.3^a^	nd	5.28 ± 0.14^h^	428.9 ± 68.1^a^	580.6 ± 66^c^	20.49 ± 0.13^ef^	2800 ± 474^fg^	
Coriander × Coconut fiber	76.75 ± 10.6^bc^	89.20 ± 17.0^a^	4113 ± 728^h^	66.64 ± 3.34b	41.98 ± 4.55^a^	20.73 ± 0.10^a^	560.7 ± 69.8^a^	102.25 ± 3.5^ef^	nd	769.1 ± 44.9^a^	50.4 ± 16^g^	644.4 ± 139^ab^	641 ± 139.2^ab^	69.25 ± 6.9^b^	nd	4.66 ± 0.15^h^	235.4 ± 18.0^b^	617.5 ± 98^c^	20.95 ± 0.24^ab^	5427 ± 996^cde^	13.55 ± 1.93^efg^
Coriander × Peat moss	84.70 ± 1.4^b^	86.24 ± 3.3^a^	4822 ± 852^gh^	66.81 ± 2.72^b^	29.78 ± 0.47^c^	20.66 ± 0.09^ab^	549.5 ± 28.6^a^	109.85 ± 5.6^e^	nd	288.1 ± 5.8^b^	134.8 ± 54^g^	690.8 ± 103^a^	689 ± 102.8^a^	53.83 ± 6.2^c^	nd	4.57 ± 0.18^h^	434.0 ± 40.3^a^	945.6 ± 84^a^	20.2 ± 0.11^gh^	5406 ± 530^cde^	14.44 ± 0.43^defg^
Kohlrabi × Agave fiber	23.73 ± 0.3^gh^	28.60 ± 0.5^cd^	5846 ± 164^gh^	22.95 ± 0.11^e^	19.72 ± 0.02^d^	19.49 ± 0.00^f^	76.37 ± 2.8^de^	62.60 ± 1.1^g^	15.96 ± 1.75^e^	130.8 ± 4.1^ef^	228.3 ± 11^f^	127.8 ± 3c	84.6 ± 18.5^ef^	4.22 ± 0.1^d^	8.13 ± 0.19h	22.14 ± 0.71^g^	150.5 ± 3.8^cd^	nd	20.5 ± 0.03^def^	6158 ± 11^cd^	13.05 ± 0.13^fg^
Kohlrabi × Capillary mat	59.64 ± 1.9^d^	36.52 ± 1.0^c^	11974 ± 601^b^	24.77 ± 0.21^de^	19.95 ± 0.02^d^	19.54 ± 0.01^f^	97.49 ± 4.3^de^	159.55 ± 9.4^d^	33.24 ± 0.80^c^	138.8 ± 3.9^ef^	258.4 ± 4^ef^	100.2 ± 3^c^	154 ± 3.7^ef^	4.41 ± 0.1^d^	13.89 ± 0.69^f^	30.48 ± 1.64^ef^	262.4 ± 8.1^b^	nd	20.4 ± 0.04^fgh^	4181 ± 128^ef^	17.59 ± 0.59^bc^
Kohlrabi × Cellulose sponge	30.18 ± 0.9^efg^	29.33 ± 0.6^cd^	5724 ± 200^gh^	22.50 ± 0.16^e^	19.81 ± 0.02^d^	19.55 ± 0.01^f^	68.37 ± 3.2^de^	158.42 ± 3.7^d^	33.94 ± 0.74^c^	213.5 ± 3.5^c^	343.2 ± 18^de^	109.8 ± 5c	207 ± 10.0^def^	4.28 ± 0.1^d^	10.94 ± 0.50^g^	34.65 ± 1.42^de^	245.5 ± 7.0^b^	nd	20.7 ± 0.04^bcdef^	7852 ± 202^b^	
Kohlrabi × Coconut fiber	17.01 ± 0.5^h^	12.96 ± 0.4^d^	4176 ± 223^h^	21.86 ± 0.27^e^	19.49 ± 0.00^d^	19.50 ± 0.00^f^	55.75 ± 2.1^e^	78.18 ± 2.8^g^	19.56 ± 1.25^e^	166.8 ± 2.9^de^	357.3 ± 12^d^	128.4 ± 4c	28.1 ± 0.7^f^	4.58 ± 0.1^d^	9.65 ± 0.23^gh^	28.82 ± 0.94^f^	197.8 ± 4.9^bc^	nd	20.7 ± 0.06^bcde^	6601 ± 41^bcd^	11.96 ± 0.26^g^
Kohlrabi × Peat moss	71.12 ± 1.4^c^	83.79 ± 1.4^a^	6162 ± 134^fg^	22.87 ± 0.13^e^	19.65 ± 0.01^d^	19.64 ± 0.01^f^	96.79 ± 2.9^de^	315.19 ± 15.9^c^	24.66 ± 1.74^d^	199.7 ± 7.4^cd^	271.3 ± 5^def^	104.3 ± 2^c^	350 ± 10.6^cd^	4.52 ± 0.0^d^	11.34 ± 0.42^g^	36.16 ± 1.80^d^	253.8 ± 7.0^b^	nd	20.8 ± 0.03^bc^	15467 ± 660^a^	23.53 ± 0.64^a^
Pak choi × Agave fiber	36.53 ± 0.7^ef^	35.70 ± 0.7^c^	15750 ± 462^a^	30.84 ± 0.22^d^	19.71 ± 0.01^d^	20.33 ± 0.03^d^	179.5 ± 2.2^c^	105.13 ± 5.0^ef^	17.40 ± 0.50^e^	162.3 ± 5.9^de^	762.1 ± 31c	163.0 ± 2^c^	129 ± 11.2^ef^	10.22 ± 0.1^d^	28.96 ± 0.27^e^	38.71 ± 1.45^d^	68.2 ± 1.2^d^	nd	20.8 ± 0.03^bc^	753 ± 35^h^	18.33 ± 0.49^b^
Pak choi × Capillary mat	31.80 ± 0.4^efg^	28.16 ± 0.6^cd^	9093 ± 256^cde^	27.33 ± 0.38^de^	19.54 ± 0.00^d^	20.30 ± 0.06^d^	145.9 ± 5.1^cd^	77.46 ± 1.6^g^	32.56 ± 1.99c	120.0 ± 11.6^f^	1224.0 ± 44^a^	122.8 ± 7^c^	129 ± 18.3^ef^	11.23 ± 0.4^d^	58.07 ± 1.53^b^	66.64 ± 4.02^c^	76.4 ± 1.9^d^	nd	20.8 ± 0.03^bcd^	4261 ± 157^ef^	15.57 ± 0.25^cdef^
Pak choi × Cellulose sponge	24.80 ± 0.2^fgh^	36.49 ± 6.6^c^	10697 ± 296^bc^	25.45 ± 0.50^de^	19.81 ± 0.01^d^	20.13 ± 0.01^e^	129.8 ± 3.2^cde^	577.69 ± 14.7^b^	35.29 ± 2.05^c^	78.0 ± 5.0^g^	900.9 ± 65^b^	110.7 ± 1^c^	219 ± 7.6^de^	11.46 ± 0.9^d^	33.77 ± 1.61^d^	66.42 ± 2.05^c^	150.1 ± 6.1^cd^	nd	20.7 ± 0.04^cdef^	3100 ± 140^fg^	16.26 ± 0.42^bcde^
Pak choi × Coconut fiber	28.66 ± 0.6^fgh^	27.68 ± 0.6^cd^	7580 ± 253^ef^	25.05 ± 0.46^de^	19.49 ± 0.00^d^	20.42 ± 0.02^cd^	72.90 ± 1.7^de^	103.81 ± 3.7^ef^	42.32 ± 1.82^b^	153.5 ± 6.7^ef^	678.4 ± 16^c^	141.5 ± 8^c^	112 ± 4.0^ef^	11.85 ± 0.6^d^	69.36 ± 1.26^a^	96.72 ± 3.75^a^	80.0 ± 2.6^d^	nd	21.2 ± 0.05^a^	4912 ± 1308^de^	14.20 ± 1.19^defg^
Pak choi × Peat moss	41.77 ± 0.4^e^	65.44 ± 6.4^b^	2009 ± 87^i^	20.83 ± 0.10^e^	19.41 ± 0.00^d^	20.03 ± 0.02^e^	60.23 ± 2.0^e^	709.93 ± 13.4^a^	50.24 ± 3.84^a^	53.0 ± 3.0^g^	747.3 ± 20^c^	103.9 ± 1c	144 ± 2.6^ef^	9.25 ± 0.6d	37.34 ± 1.11c	91.17 ± 1.51^b^	102.3 ± 10.3^d^	nd	20.4 ± 0.04^efg^	2454 ± 56^g^	6.76 ± 0.13^h^
Significance																					
Microgreens species (M)	***	***	***	***	***	***	***	***	***	***	***	***	***	***	***	***	***	***	***	***	***
Substrate (S)	***	***	***	***	***	**	**	***	***	***	***	**	***	***	***	***	***	***	***	***	***
M × S	***	***	***	***	***	***	**	***	***	***	***	***	***	***	***	***	**	***	***	***	***

ns, *, **, *** Nonsignificant or significant at *p* ≤ 0.05, 0.01, and 0.001, respectively. Different superscript letters (a–h) within each column indicate significant differences according to Duncan’s multiple-range test (*p* = 0.05).

## References

[1] Poiroux-Gonord F., Bidel L.P., Fanciullino A.L., Gautier H., Lauri-Lopez F., Urban L. (2010). Health benefits of vitamins and secondary metabolites of fruits and vegetables and prospects to increase their concentrations by agronomic approaches. J. Agric. Food Chem..

[2] Kyriacou M.C., Rouphael Y. (2018). Towards a new definition of quality for fresh fruits and vegetables. Sci. Hortic..

[3] Rouphael Y., Kyriacou M.C., Petropoulos S.A., de Pascale S., Colla G. (2018). Improving vegetable quality in controlled environments. Sci. Hortic..

[4] Xiao Z., Lester G.E., Luo Y., Wang Q. (2012). Assessment of vitamin and carotenoid concentrations of emerging food products: Edible microgreens. J. Agric. Food Chem..

[5] Xiao Z., Lester G.E., Park E., Saftner R.A., Luo Y., Wang Q. (2015). Evaluation and correlation of sensory attributes and chemical compositions of emerging fresh produce: Microgreens. Postharvest Biol. Technol..

[6] Di Gioia F., Mininni C., Santamaria P., di Gioia F., Santamaria P. (2015). How to grow microgreens. Microgreens: Novel Fresh and Functional Food to Explore All the Value of Biodiversity.

[7] Pinto E., Almeida A.A., Aguiar A.A., Ferreira I.M.P.L.V.O. (2015). Comparison between the mineral profile and nitrate content of microgreens and mature lettuces. J. Food Comp. Anal..

[8] Choe U., Yu L.L., Wang T.T.Y. (2018). The science behind microgreens as an exciting new food for the 21st century. J. Agric. Food Chem..

[9] Lenzi A., Orlandini A., Bulgari R., Ferrante A., Bruschi P. (2019). Antioxidant and mineral composition of three wild leafy species: A comparison between microgreens and baby greens. Foods.

[10] Kyriacou M.C., Rouphael Y., di Gioia F., Kyratzis A., Serio F., Renna M., de Pascale S. (2016). Micro-scale vegetable production and the rise of microgreens. Trends Food Sci. Technol..

[11] Kyriacou M.C., de Pascale S., Kyratzis A., Rouphael Y. (2017). Microgreens as a component of space life support systems: A cornucopia of functional food. Front. Plant Sci..

[12] Brazaitytė A., Sakalauskiené S., Samuoliené G., Jankauskienė J., Viršilė A., Novičkovas A., Sirtautas R., Miliauskienė J., Vaštakaitė V., Dabašinskas L. (2015). The effects of LED illumination spectra and intensity on carotenoid content in *Brassicaceae* microgreens. Food Chem..

[13] Kyriacou M.C., El-Nakhel C., Graziani G., Pannico A., Soteriou G.A., Giordano M., Ritieni A., de Pascale S., Rouphael Y. (2019). Functional quality in novel food sources: Genotypic variation in the nutritive and phytochemical composition of thirteen microgreens species. Food Chem..

[14] Kyriacou M.C., El-Nakhel C., Pannico A., Graziani G., Soteriou G.A., Giordano M., Zarrelli A., Ritieni A., De Pascale S., Rouphael Y. (2019). Genotype-specific modulatory effects of select spectral bandwidths on the nutritive and phytochemical composition of microgreens. Front. Plant Sci..

[15] Samuoliene G., Brazaitytė A., Viršilė A., Jankauskienė J., Sakalauskiene S., Duchovskis P. (2016). Red light-dose or wavelength-dependent photoresponse of antioxidants in herb microgreens. PLoS ONE.

[16] Samuolienė G., Viršilė A., Brazaitytė A., Jankauskienė J., Sakalauskienė S., Vaštakaitė V., Novičkovas A., Viškelienė A., Sasnauskas A., Duchovskis P. (2017). Blue light dosage affects carotenoids and tocopherols in microgreens. Food Chem..

[17] Craver J.K., Gerovac J.R., Lopez R.G., Kopsell D.A. (2017). Light intensity and light quality from sole-source light-emitting diodes impact phytochemical concentrations within *Brassica* microgreens. J. Am. Soc. Hortic. Sci..

[18] Vaštakaitė V., Viršilė A., Brazaitytė A., Samuolienė G., Jankauskienė J., Novičkovas A., Duchovskis P. (2017). Pulsed light emitting diodes for a higher phytochemical level in microgreens. J. Agric. Food Chem..

[19] Alrifai O., Hao X., Marcone M.F., Tsao R. (2019). Current review of the modulatory effects of LED lights on photosynthesis of secondary metabolites and future perspectives of microgreen vegetables. J. Agric. Food Chem..

[20] De la Fuente B., López-García G., Máñez V., Alegría A., Barberá R., Cilla A. (2019). Evaluation of the bioaccessibility of antioxidant bioactive compounds and minerals of four genotypes of *Brassicaceae* Microgreens. Foods.

[21] Xiao Z., Rausch S.R., Luo Y., Sun J., Yu L., Wang Q., Chen P., Yu L., Stommel J.R. (2019). Microgreens of *Brassicaceae*: Genetic diversity of phytochemical concentrations and antioxidant capacity. LWT.

[22] Di Gioia F., De Bellis P., Mininni C., Santamaria P., Serio F. (2017). Physicochemical, agronomical and microbiological evaluation of alternative growing media for the production of rapini (*Brassica rapa* L.) microgreens. J. Sci. Food Agric..

[23] Muchjajib U., Muchjajib S., Suknikom S., Butsai J. (2015). Evaluation of organic media alternatives for the production of microgreens in Thailand. Acta Hortic..

[24] Rouphael Y., Colla G., Giordano M., El-Nakhel C., Kyriacou M.C., de Pascale S. (2017). Foliar applications of a legume-derived protein hydrolysate elicit dose-dependent increases of growth, leaf mineral composition, yield and fruit quality in two greenhouse tomato cultivars. Sci. Hortic..

[25] Lichtenthaler H.K., Buschmann C. (2001). Chlorophylls and carotenoids: Measurement and characterization by UV-VIS spectroscopy. Curr. Protoc. Food Anal. Chem..

[26] Kampfenkel K., van Montagu M., Inzé D. (1995). Extraction and determination of ascorbate and dehydroascorbate from plant tissue. Anal. Biochem..

[27] Kim H.J., Fonseca J.M., Choi J.H., Kubota C., Kwon D.Y. (2008). Salt in irrigation water affects the nutritional and visual properties of Romaine lettuce (*Lactuca sativa* L.). J. Agric. Food Chem..

[28] Llorach R., Martínez-Sánchez A., Tomás-Barberán F.A., Gil M.I., Ferreres F. (2008). Characterisation of polyphenols and antioxidant properties of five lettuce varieties and escarole. Food Chem..

[29] Abad M., Noguera P., Bures S. (2011). National inventory of organic wastes for use as growing media for ornamental potted plant production: Case study in Spain. Bioresour. Technol..

[30] Barrett G.E., Alexander P.D., Robinson J.S., Bragg N.C. (2016). Achieving environmentally sustainable growing media for soilless plant cultivation systems—A review. Sci. Hortic..

[31] Savvas D. (2007). Modern developments in the use of inorganic media for greenhouse vegetable and flower production. Acta Hort..

[32] Schmilewski G. (2008). The role of peat in assuring the quality of growing media. Mires Peat.

[33] Sánchez-Monedero M.A., Roig A., Cegarra J., Bernal M.P., Noguera P., Abad M., Antón A. (2004). Composts as media constituents for vegetable transplant production. Compost Sci. Utilization.

[34] Shannon M.C., Grieve C.M. (1998). Tolerance of vegetable crops to salinity. Sci. Hortic..

[35] Okkaoğlu H., Sönmez Ç., Şimşek A.Ö., Bayram E. (2015). Effect of salt stress on some agronomical characteristics and essential oil content of coriander (*Coriandrum sativum* L.) cultivars. J. Appl. Biol. Sci. JABS.

[36] Bulgari R., Baldi A., Ferrante A., Lenzi A. (2017). Yield and quality of basil, Swiss chard, and rocket microgreens grown in a hydroponic system. N. Z. J. Crop Hortic. Sci..

[37] Carillo P., Raimondi G., Kyriacou M.C., Pannico A., El-Nakhel C., Cirillo V., Colla G., de Pascale S., Rouphael Y. (2019). Morpho-physiological and homeostatic adaptive responses triggered by omeprazole enhance lettuce tolerance to salt stress. Sci. Hortic..

[38] Santamaria P. (2006). Nitrate in vegetables: Toxicity, content, intake and EC regulation. J. Sci. Food Agric..

[39] Colla G., Kim H.J., Kyriacou M.C., Rouphael Y. (2018). Nitrate in fruits and vegetables. Sci. Hortic..

[40] Raviv M., Wallach R., Silber A., Bar-Tal A. (2002). Substrates and their analysis. Hydroponic Prod. Veg. Ornam..

[41] (2006). Commission Regulation (EC) No. 1881/2006 of 19 December Setting Maximum Levels for Certain Contaminants in Foodstuffs.

[42] EFSA (2008). Opinion of the scientific panel on contaminants in the food chain on a request from the European commission to perform a scientific risk assessment on nitrate in vegetables. EFSA J..

[43] Gharibzahedi S.M.T., Jafari S.M. (2017). The importance of minerals in human nutrition bioavailability, food fortification, processing effects and nanoencapsulation. Trends Food Sci. Technol..

[44] Levander O.A. (1990). Fruit and vegetable contributions to dietary mineral intake in human health and disease. HortScience.

[45] Xiao Z., Codling E.E., Luo Y., Nou X., Lester G.E., Wang Q. (2016). Microgreens of *Brassicaceae*: Mineral composition and content of 30 varieties. J. Food Compos. Anal..

[46] Neugart S., Baldermann S., Hanschen F.S., Klopsch R., Wiesner-Reinhold M., Schreiner M. (2018). The intrinsic quality of brassicaceous vegetables: How secondary plant metabolites are affected by genetic, environmental, and agronomic factors. Sci. Hortic..

[47] Young A.J., Lowe G.M. (2001). Antioxidant and prooxidant properties of carotenoids. Arch. Biochem. Biophys..

[48] Kvansakul J., Rodriguez-Carmona M., Edgar D.F., Barker F.M., Koepcke W., Schalch W., Barbur J.L. (2006). Supplementation with the carotenoids lutein or zeaxanthin improves human visual performance. Ophthalmic Physiol. Opt..

[49] Saltveit M.E., Choi Y.J., Tomas-Barberan F.A. (2005). Involvement of components of the phosopholipid-signalling pathway in wound-induced phenylpropanoid metabolism in lettuce (*Lactuca sativa*) leaf tissue. Physiol. Plant..

[50] Vallejo F., García-Viguera C., Tomás-Barberán F.A. (2003). Changes in broccoli (*Brassica oleracea* L. var. *italica)* health-promoting compounds with inflorescence development. J. Agric. Food Chem..

[51] Boerjan W., Ralph J., Baucher M. (2003). Lignin biosynthesis. Annu. Rev. Plant Biol..

[52] Onakpoya I.J., Spencer E.A., Thompson M.J., Heneghan C.J. (2014). The effect of chlorogenic acid on blood pressure: A systematic review and meta-analysis of randomized clinical trials. J. Hum. Hypertens..

[53] Tajik N., Takik M., Mack I., Enck P. (2017). The potential effects of chlorogenic acid, the main phenolic components in coffee, on health: A comprehensive review of the literature. Eur. J. Nutr..

[54] Whitaker B.D., Stommel J.R. (2003). Distribution of hydroxycinnamic acid conjugates in fruit of commercial eggplant (*Solanum melongena* L.) cultivars. J. Agric. Food Chem..

[55] Santos J.S., Escher G.B., do Carmo M.V., Azevedo L., Marques M.B., Daguer H., Oh W.Y. (2020). A new analytical concept based on chemistry and toxicology for herbal extracts analysis: From phenolic composition to bioactivity. Food Res. Int..

[56] Alfaifi M., Alsayari A., Gurusamy N., Louis J., Eldin Elbehairi S., Venkatesan K., Alboushnak H. (2020). Analgesic, anti-Inflammatory, cytotoxic activity screening and UPLC-PDA-ESI-MS metabolites determination of bioactive fractions of *Kleinia pendula*. Molecules.

[57] Terao J., Yamaguchi S., Shirai M., Miyoshi M., Moon J.H., Oshima S., Inakuma T., Tsushida T., Kato Y. (2001). Protection by quercetin and quercetin 3-O-β-D-glucuronide of peroxynitrite-induced antioxidant consumption in human plasma low-density lipoprotein. Free Radic. Res..

[58] Cartea M.E., Francisco M., Soengas P., Velasco P. (2011). Phenolic compounds in *Brassica* vegetables. Molecules.

[59] Olsen H., Aaby K., Borge G.I. (2009). Characterization and quantification of flavonoids and hydroxycinnamic acids in curly kale (*Brassica oleracea* L. convar. *acephala* var. *sabellica)* by Hplc-Dadesi-Msn. J. Agric. Food Chem..

[60] Lesjak M., Beara I., Simin N., Pintać D., Majkić T., Bekvalac K., Mimica-Dukić N. (2018). Antioxidant and anti-inflammatory activities of quercetin and its derivatives. J. Func. Food.

[61] El-Nakhel C., Pannico A., Kyriacou M.C., Giordano M., de Pascale S., Rouphael Y. (2019). Macronutrient deprivation eustress elicits differential secondary metabolites in red and green-pigmented butterhead lettuce grown in closed soilless system. J. Sci. Food Agric..

